# Inflammation-Driven Breast Tumor Cell Plasticity: Stemness/EMT, Therapy Resistance and Dormancy

**DOI:** 10.3389/fonc.2020.614468

**Published:** 2021-01-26

**Authors:** Tamir Baram, Linor Rubinstein-Achiasaf, Hagar Ben-Yaakov, Adit Ben-Baruch

**Affiliations:** George S. Wise Faculty of Life Sciences, The Shmunis School of Biomedicine and Cancer Research, Tel Aviv University, Tel Aviv, Israel

**Keywords:** cytokines/chemokines, dormancy, epithelial-to-mesenchymal transition, inflammation, macrophages, stemness, therapy resistance, tumor cell plasticity

## Abstract

Cellular heterogeneity poses an immense therapeutic challenge in cancer due to a constant change in tumor cell characteristics, endowing cancer cells with the ability to dynamically shift between states. Intra-tumor heterogeneity is largely driven by cancer cell plasticity, demonstrated by the ability of malignant cells to acquire stemness and epithelial-to-mesenchymal transition (EMT) properties, to develop therapy resistance and to escape dormancy. These different aspects of cancer cell remodeling are driven by intrinsic as well as by extrinsic signals, the latter being dominated by factors of the tumor microenvironment. As part of the tumor milieu, chronic inflammation is generally regarded as a most influential player that supports tumor development and progression. In this review article, we put together recent findings on the roles of inflammatory elements in driving forward key processes of tumor cell plasticity. Using breast cancer as a representative research system, we demonstrate the critical roles played by inflammation-associated myeloid cells (mainly macrophages), pro-inflammatory cytokines [such as tumor necrosis factor α (TNFα) and interleukin 6 (IL-6)] and inflammatory chemokines [primarily CXCL8 (interleukin 8, IL-8) and CXCL1 (GROα)] in promoting tumor cell remodeling. These inflammatory components form a common thread that is involved in regulation of the three plasticity levels: stemness/EMT, therapy resistance, and dormancy. In view of the fact that inflammatory elements are a common denominator shared by different aspects of tumor cell plasticity, it is possible that their targeting may have a critical clinical benefit for cancer patients.

## Introduction

The global effort to develop improved therapies for cancer patients is an enduring task, partly due to tumor cell heterogeneity that characterizes many different cancer types. The reasons for heterogeneity are diverse and include among others the constant transition of tumor cells between different phenotypic and functional states. Assembled together under the term “cancer cell plasticity”, various types of transition events take place at primary tumors and in metastatic foci (1–8).

Cancer cell heterogeneity and plasticity are well-documented in breast cancer (BC), where they contribute to immense mechanistic and functional complexity, and have cardinal therapeutic implications (4, 9, 10). As they manifest inter-tumor heterogeneity, breast tumors are categorized in four groups that are differently treated in the clinic, based on the expression of estrogen receptors (ERs), progesterone receptors (PRs) and HER2: luminal-A cancers, where tumors express ER/PR and are HER2-negative (HER2−); luminal-B tumors that are positive for ER/PR and demonstrate HER2 amplification (HER2+) or are ki67-high; HER2+ tumors that do not express ER/PR and demonstrate HER2 over-expression; and triple-negative BCs (TNBCs) that lack the expression of the three receptors: ER, PR and HER2 (11–13).

In parallel to inter-tumor heterogeneity, intra-tumor heterogeneity is also highly apparent in BC. Breast tumors can include cancer stem cells (CSCs) and non-CSCs as well (4, 9, 10); some of the cancer cells undergo epithelial-to-mesenchymal transition (EMT) while others do not (10); under certain conditions, cells that develop acquired resistance to different types of therapy are present among the cancer cells (4, 14); and in addition, cancer cells can gain the ability to remain dormant as single or clustered micro-metastasizing cells or exhibit through different modifications the ability to exit dormancy (4, 15).

These different heterogeneity facets are often linked to each other, as seen for example by the connection between CSC and EMT states, by the fact that CSCs are highly resistant to chemotherapy and by the regulation of CSC/EMT and dormancy by chemotherapy (1–4). Also, they exemplify the high degree of plasticity that characterizes cancer cells. Non-CSCs can turn to CSCs and *vice versa* (1, 5, 6); cells that have undergone EMT can more efficiently metastasize and then colonize better the metastatic niche if they have completed the opposite process of mesenchymal-to-epithelial transition (MET) (5); therapy-resistance is subject to alterations that have a strong impact on the well-being of patients and their survival, and tumor cells can adapt to stress by entering a dormant phase but can also escape dormancy when conditions change (2, 3, 6).

This dynamic remodeling of the cancer cells depends on cell-autonomous traits (*e.g.* epigenetics, metabolism, endoplasmic reticulum stress) but in parallel the tumor microenvironment (TME) has substantial ability to shape the phenotypes and functions of the cancer cells and thus dictates the degree of cancer heterogeneity and plasticity (16). In this context, major roles were recently attributed to immune/inflammatory cells and to the factors that mediate their activities (17, 18). The diversity of immune cells and the balance between the acquired immunity arm and the inflammatory arm have prominent impacts on the fate of the tumor and its progression. When acquired immunity is concerned, it is well known that specific cell types, like T helper 1 (Th1) cells and cytotoxic T cells (CTLs) are key players in immune surveillance and their activities may lead to cancer cell eradication; these effects may be strengthened by immunotherapies (*e.g.*, those directed to inhibitory immune checkpoint molecules like PD-1, PD-L1, and CTLA-4) and contribute to tumor suppression (19, 20). However, acquired immunity may also have opposing effects, as demonstrated for example by the ability of Th1-derived interferon *γ* (IFN*γ*) to up-regulate inhibitory checkpoint molecules (21), while Th2 cells can release cytokines that divert macrophages to an M2 phenotype that supports tumor progression (22). As expected, different aspects of cancer cell plasticity were shown to be regulated by cells and factors of acquired immunity [*e.g.*, (17, 18, 23, 24)]; however, in view of the many facets of acquired immunity in malignancy, this topic will not be addressed in depth in this review.

**Figure 1:**
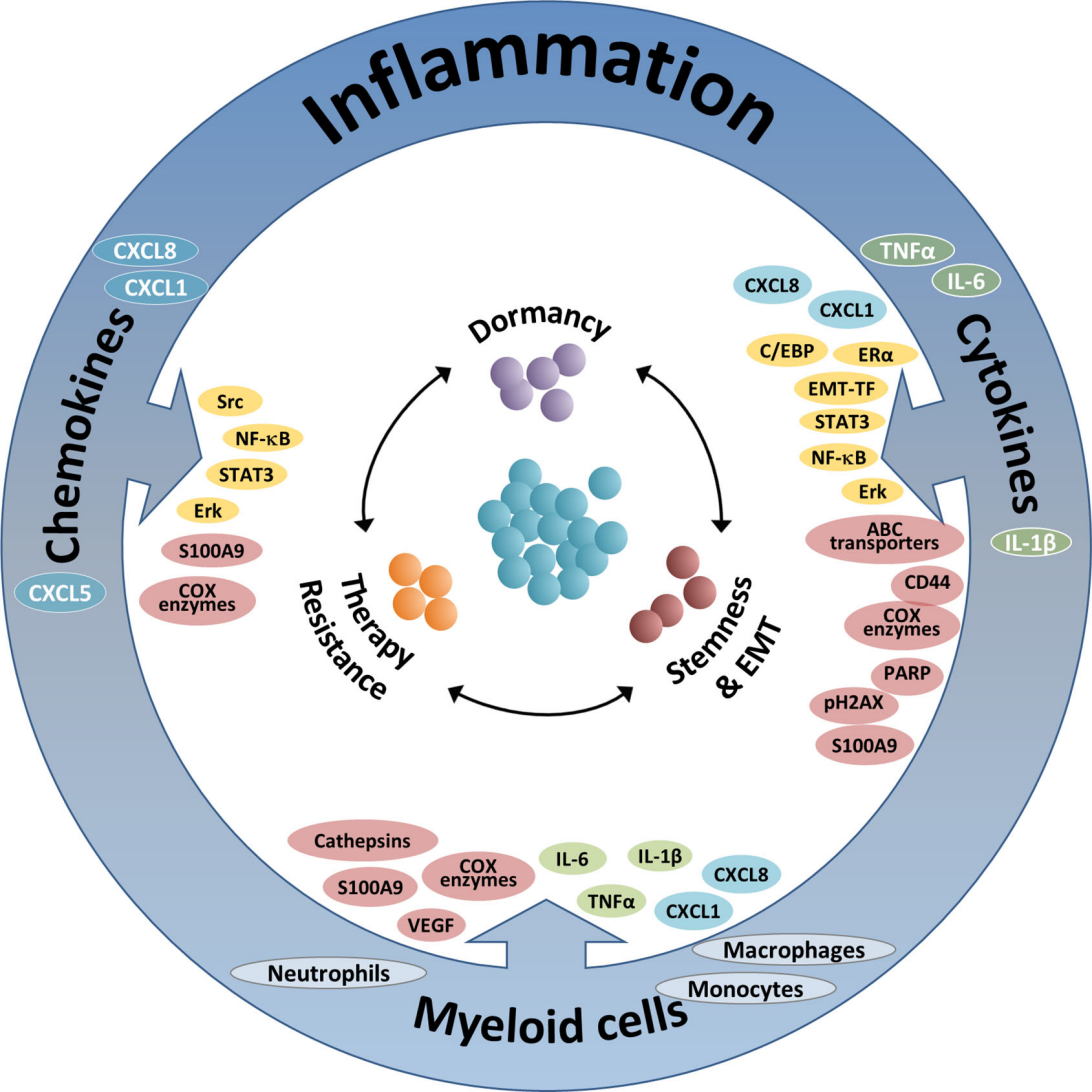
The integrated activities of pro-inflammatory cells and soluble pro-inflammatory factors in controlling tumor cell plasticity. The Figure demonstrates the roles of three inflammatory axes in regulating key aspects of cancer cell plasticity: stemness/EMT, therapy resistance and dormancy (see also **Tables 1**–**3**). Based on the findings summarized in this review, it is proposed that therapeutic modalities that target such key inflammatory elements will affect simultaneously several modes of malignant cell plasticity and will therefore provide improved approaches of cancer therapy. The three inflammatory axes addressed in this manuscript include: (1) Inflammation-associated myeloid cells: Here, the main contributors to regulation of tumor cell plasticity are macrophages and monocytes, and also neutrophils. These cells control tumor cell remodeling by forming physical contacts and also by exchanging soluble mediators with the cancer cells. The major elements involved in the activities of myeloid cells include: In green—Pro-inflammatory cytokines; In cyan—Inflammatory chemokines; In brown—Other intracellular components. (2) Pro-inflammatory cytokines: In this context, major roles are attributed mainly to the pro-inflammatory cytokines TNFα and IL-6 (and to some extent also IL-1β), whose activities are mediated mainly by activation of transcription factors (TFs) and up-regulation of inflammatory chemokines. The main elements involved in this axis include: In yellow—Inflammation-related TFs and other TFs; In cyan—Inflammatory chemokines; In brown - Other intracellular components. (3) Inflammatory chemokines: Here, mainly chemokines of the ELR+ CXC sub-group are implicated, primarily CXCL8, CXCL1 and CXCL5; other chemokines such as CCL2 and CCL5 are also involved, as discussed in brief in the manuscript (not shown). In this setting, the major elements involved include: In yellow—Inflammation-related TFs and other TFs; In brown—Other intracellular components.

In parallel to acquired immunity, myeloid cells and pro-inflammatory products exert a large variety of effects that contribute to increased metastasis and reduced survival, mainly at more advanced stages of tumor progression. Regarded as “The seventh hallmark of cancer”, cancer-related inflammation has a very strong impact on disease progression, contributing to tumor development and metastasis (25–28). In view of their critical functions at the TME and their significant impacts on disease course, it is no surprise that inflammatory elements also regulate many cardinal aspects of tumor cell remodeling, as discussed and illustrated in this review article (summarized in **Figure 1** and **Tables 1**–**3**).

**Table 1:** Inflammation-driven cellular and molecular mechanisms regulating stemness and EMT.

STEMNESS and EMT	
Inflammatory element involved	Main observations
**Myeloid cells**	
Monocytic cells/ Macrophages/TAMs	* *Via* physical contacts with cancer cells and through secreted factors, they increased CSC proportions, as well as EMT and migration. * The activities of the myeloid cells were mediated by or connected to soluble factors such as TNFα, IL-6 and ELR+ CXC chemokines (*e.g.*, CXCL8 and CXCL1).
**Soluble and cellular mediators**	
TNFα, IL-6, IL-1β, ELR+ CXC chemokines	* Inflammatory cytokines sustained CD90+ CSCs in tumor cells, and had a profound ability to increase tumor cell stemness, EMT and migration. * The soluble mediators induced predominantly the activation of NF-kB and STAT3. Activation of HER2/EGFR-Src was also described. * Regulation of EMT-related transcription factors was induced by the soluble mediators, or was linked to their activity.

The table summarizes the cellular and molecular components that are involved in inflammation-driven regulation of stemness and EMT. To enable a unifying view of the findings described in different research systems, the table focuses on the major observations described in the text, related to the functions of myeloid cells, pro-inflammatory cytokines and ELR+ CXC chemokines (without CC chemokines).

**Table 2:** Inflammation-driven cellular and molecular mechanisms regulating therapy resistance.

THERAPY RESISTANCE	
Inflammatory element involved	Main observations
**Chemoresistance**
**Myeloid cells**
Macrophages, CD11b+Gr1+ cells	* Myeloid cells released soluble factors that promoted chemoresistance, with evidence to increased proportions of chemoresistant ALDH+ CSCs. * The mediators involved in chemoresistance included mainly TNFα, cathepsins and S100A9.
**Soluble and cellular mediators**
TNFα, mTNFα, ELR+ CXC chemokines, cathepsins, S100A9	* TNFα was directly active in promoting chemoresistance, in its soluble and membrane forms. * TNFα has induced the expression of chemokines that recruited myeloid cells to tumors. * ELR+ chemokines directly promoted chemoresistance, for example by elevating tumor cell viability in a S100A9-dependent manner. * Cathepsins protected the cancer cells from chemotherapy. * The molecular elements involved in chemoresistance included primarily NF-kB and STAT3 activation, and also activation of C/EBP and of the Lin-28B-Let7-HMGA2 axis. * Inhibition of pH2AX expression and elevated expression of PARP were involved in chemoresistance, as well as elevated expression of ABC transporters.
**Endocrine resistance**
**Myeloid cells**
Macrophages	* Through the release of soluble mediators, macrophages have led to resistance to estrogen withdrawal and to tamoxifen resistance. * The soluble mediators involved included mainly TNFα, IL-6 and chemokines.
**Soluble and cellular mediators**
TNFα, IL-6, ELR+ CXC chemokines	* The soluble mediators reduced the expression of ERα or have led to its constitutive activation. * The molecular mechanisms included activation of the NF-*k*B and STAT3 pathways, down-regulation of FOXO3a and involvement of COX-2.

The table summarizes the cellular and molecular components that are involved in inflammation-driven regulation of therapy resistance (addressing here only chemotherapy and endocrine therapy) and dormancy. To enable a unifying view of the findings described in different research systems, the Table focuses on the major observations described in the text, related to the functions of myeloid cells, pro-inflammatory cytokines and ELR+ CXC chemokines (without CC chemokines).

**Table 3:** Inflammation-driven cellular and molecular mechanisms regulating tumor cell dormancy.

DORMANCY	
Inflammatory element involved	Main observations
**Myeloid cells**	
Macrophages, monocytes, neutrophils	* Myeloid cells were connected to tumor recurrence following surgery and to exit from dormancy. * Myeloid cells acted in this respect *via* soluble mediators like TNFα, IL-1β and VEGF-A, and through COX enzymes.
**Soluble and cellular mediators**	
TNFα, IL-1β, IL-6, ELR+ CXC chemokines, COX enzymes	* Inflammatory cytokines increased the proliferation of dormant cells, including at the bone niche. * TNFα induced chemokine expression *via* the IKK-NF-*k*B pathway, and together with other pro-inflammatory cytokines has led to increased tumor cell proliferation and exit from dormancy. * STAT3 was also found to be a key regulator of cytokine activities in dormancy (its activities depended on the ligand used). * Stimulation of CXCR2 by its cognate ELR+ chemokines has led to Erk signaling; it increased cancer cell proliferation under quiescent conditions and promoted emergence from chemotherapy-induced dormancy. * COX-2 has led to increased aromatase expression, leading to elevated ERα activity and tumor cell proliferation.

The table summarizes the cellular and molecular components that are involved in inflammation-driven regulation of cancer cell dormancy. To enable a unifying view of the findings described in different research systems, the Table focuses on the major observations described in the text, related to the functions of myeloid cells, pro-inflammatory cytokines and ELR+ CXC chemokines (without CC chemokines).

Herein, we focus on the inflammatory arena and its impacts on cancer cell plasticity. To demonstrate the activities of inflammatory players in controlling tumor cell remodeling, we highlight their roles in dictating the dynamic nature of tumor cells at the following three major levels of plasticity: (1) Stemness and EMT; (2) Resistance to different types of therapy; (3) Entry to and exit from dormancy (1–10).

In addressing these three plasticity-related topics, we hereby focus on breast malignancy in order to exemplify the roles of three key inflammatory axes:

**(1) Inflammation-associated myeloid cells:** Breast tumors are usually characterized by pronounced chronic inflammation and are enriched with different types of myeloid cells. Here, major roles are attributed to tumor-associated macrophages (TAMs) in promoting cancer progression (22, 29). TAMs are typically regarded as alternatively-activated M2 macrophages, but under certain conditions macrophages with a classically-activated M1 phenotype can also have significant roles in promoting cancer progression (30–32).

**(2) Pro-inflammatory cytokines:** Here, we put major emphasis on tumor necrosis factor α (TNFα) and interleukin 6 (IL-6) and the canonical transcription factors that mediate their activities: NF-*κ*B and STAT3, respectively (25, 33–35). The chronic presence of TNFα in tumors strongly enhances tumor progression (36–40); in parallel, IL-6 is also considered a strong tumor-promoting factor (41–43). These elements are well characterized for their ability to enhance tumor progression in many malignancies including BC (35, 36, 38, 41–43).

**(3) Inflammatory chemokines:** Consisting of four structural sub-groups—of which the CXC and the CC are the largest—and homeostatic and/or inflammatory activities, chemokines are key players in protection against pathogens but also have cardinal roles in regulating malignancy (44–54). Inflammatory chemokines of the ELR+ CXC sub-group contribute immensely to tumor-related inflammation; they chemoattract leukocytes with pro-malignancy roles to tumors and they can also induce angiogenesis and act directly on the cancer cells, to promote their metastatic potential (44–54). Thus, in this review we focus on inflammatory ELR+ CXC chemokines, such as CXCL8 (interleukin 8, IL-8) and CXCL1 (GRO*α*), and we also address their respective receptors: CXCR2 (for both chemokines) and CXCR1 (for CXCL8) (52, 55–57). Occasionally, we also describe the involvement of other members of the family of inflammatory chemokines, such as CCL2 (MCP-1) and CCL5 (RANTES).

The different players that will be addressed in this manuscript—myeloid cells and inflammatory factors—are strongly linked to each other at many different manners, when pathogen-related conditions are concerned. For example, macrophages are a major source for TNFα, IL-6 and some of the chemokines (58, 59), and TNFα is a very strong inducer of many inflammatory chemokines (40, 60–63). Similarly, also in the tumor plasticity field, these different inflammatory components often play cooperative and simultaneous roles in promoting tumor cell stemness/EMT, therapy resistance, and dormancy in BC, as will be illustrated below.

## Inflammation-Driven Stemness and EMT in Breast Malignancy

To successfully complete the consecutive process of tumor cell dissemination, followed by seeding and growth in metastatic niches, cancer cells need to exhibit improved capabilities to cope with the obstacles they encounter along the way. When tumor heterogeneity is closely examined, it is possible to identify cells that have acquired stem cell characteristics and/or underwent EMT, thus demonstrating plasticity that contributes to their ability to better handle the challenges they face during metastasis (64–69).

BC cells clearly follow the same paradigm, demonstrating dynamic remodeling that leads to development/selection of cancer stem cells (CSCs) and of tumor cells that have undergone EMT. More so, similar to other cancer cell types, also in BC the two processes are closely linked, as manifested for example by the fact that CSCs often express EMT characteristics (67–69). Under these conditions, BC cells benefit from the advantages provided by these two properties together and express higher ability to metastasize and resist different therapeutic modalities (67–69).

In the context of cancer cell plasticity, stemness and EMT processes have a major influence on the functional state of cancer cells. Stemness in BC is mainly identified by increased presence of CD44+/CD24− cells or CD44+/CD24− low cells, and ALDH+ cells; by the expression of stemness genes and/or by the ability of the tumor cells to form mammospheres (tumor spheres). CSCs are also greatly connected to increased therapy resistance, particularly in response to chemotherapy (5, 9, 70, 71).

In parallel, EMT is characterized by the acquisition of mesenchymal morphology; reduced expression of E-cadherin; increased expression of N-cadherin, vimentin and/or fibronectin; and elevated expression of EMT regulators such as twist, snail, slug and zeb (5, 10, 70, 71). As expected, the EMT process is often accompanied by elevated migratory and invasive properties of the cancer cells (70, 71).

As illustrated below, inflammatory cells and soluble mediators have prominent roles in regulating stemness and EMT in breast cancer, often with interactions between the different players (summarized in **Figure 1** and **Table 1**).

### Inflammation-Associated Myeloid Cells Regulating Stemness and EMT

Macrophages form an important hub, converging the functions of many regulators of stemness and EMT. The impact of macrophages and of the factors they release on these remodeling processes were revealed by studies of monocytic cell co-cultures with breast tumor cells and by investigations in which cancer cells were grown in the presence of macrophage-derived conditioned media (CM). Under both conditions, of direct or indirect interactions between the cancer cells and monocytic cells, elevated levels of stemness, EMT or both were frequently mediated by inflammatory factors such as TNFα, IL-6 and/or ELR+ CXC chemokines that will be particularly mentioned in more detail later on.

For example, a research by Weinberg and his colleagues demonstrated that in BC patient biopsies, CD68+ macrophages were localized in proximity to CD90+ tumor cells, which were endowed with characteristics of CSCs as well as EMT (72). In this report, macrophages enhanced tumor initiation by CD90+ CSC cells and promoted the formation of primary tumors and of metastases (72). Of interest was the fact that tumor cell-expressed CD90 was required for generating physical contacts between the tumor cells and macrophages, and these interactions have increased the expression of IL-6, CXCL8 and granulocyte-macrophage colony stimulation factor (GM-CSF) in the cancer cells. In parallel, inflammatory cytokines sustained the levels of CSCs, and recombinant IL-6 and CXCL8 increased the formation of tumor spheres by CD90+ tumor cells (72).

When considering the type of macrophages that regulate stemness/EMT, it is interesting to note that when CM of M1 cells (mainly those derived from peripheral blood mononuclear cells, PBMCs) have been added to luminal-A BC cells, the tumor cells have acquired a CSC phenotype (elevated formation of mammospheres), EMT properties (mesenchymal morphology, reduced E-cadherin and elevated zeb1 expression) and more potent migration (73). By adding neutralizing antibodies to specific pro-inflammatory cytokines, the researchers demonstrated that TNFα, interleukin 1β (IL-1β) and IL-6 partly contributed to some of these effects. In line with the fact that M1-derived CM have led to STAT3 activation, inhibition of the Jak2-STAT3 pathway alongside with NF-*κ*B down-regulation resulted in reduced EMT properties and lower proportions of CD44+/CD24−/low CSCs (73).

In another investigation it was found that inflammatory BC (IBC) tumors were significantly infiltrated by CD163+ M2 macrophages; moreover, IBC cells in culture released chemokines that induced the recruitment of macrophages, as well as factors that induced macrophage polarization to the M2 direction (74). In this study, which has used THP-1 cells or blood monocytes, physical co-culturing of monocytic cells with cancer cells has given rise to elevated proportions of CSCs (CD44+/CD24− and ALDH+) and the cancer cells have gained some EMT characteristics (74). Some of these EMT phenotypes and tumor cell invasion were up-regulated by ELR+ CXC chemokines that were present in the culture medium of the macrophages, such as CXCL8 and CXCL1 (74). In this case, the chemokines have led to STAT3 activation in the tumor cells, which then has driven forward stemness and EMT (74).

In the same spirit, in response to CM of blood monocyte-derived macrophages, several breast tumor cell lines acquired a stronger invasive capability; in MCF-7 cells, this effect was mediated by TNFα-induced stabilization of snail, mediated by the ability of the cytokine to activate NF-*κ*B (75). Moreover, in a study using TAMs excised from a MMTV-PyMT tumor model and monocytic cells, the overall conclusion was that macrophages released CXCL1, which *via* NF-*κ*B activation has induced the transcription of the EMT regulator SOX4, leading to EMT and metastasis (76). It is interesting to note that in this study, CXCL1 was not found to induce the enrichment of CSCs (76).

### Pro-Inflammatory Cytokines Regulating Stemness and EMT

The above studies demonstrated that contacts formed between monocytic cells and BC cells, as well as factors released by macrophages had a major role in promoting stemness and EMT in BC. As mentioned, the factors involved in macrophage-mediated regulation of stemness and EMT included pro-inflammatory factors such as TNFα, IL-1β, IL-6 and the ELR+ CXC chemokines CXCL8 and CXCL1. In parallel, in other studies, these soluble factors were found to promote the proportions of CSCs and/or of cells undergoing EMT independently of macrophage-related aspects.

For example, the roles of TNFα were studied extensively in BC by using it in a recombinant form or by employing CM of TNFα-stimulated cells. In many studies, TNFα has directly induced stemness, EMT, and migration in BC cells and in non-transformed breast epithelial cells; in this regard, TNFα acted when it was used alone or when it was joined by other TME factors that often potentiated its activities, such as the EMT-inducer transforming growth factor *β* (TGF*β*) or estrogen + epidermal growth factor (EGF) (40, 62, 63, 77–88). In some of the settings, it was the extended stimulation of cells by TNFα for up to several weeks that has led to pronounced effects on stemness as well as on EMT (77–79). Mechanistically, TNFα activities were accompanied by modified expression of CSC markers (like elevated presence of CD44+/CD24− or CD44+/CD24− low cells) and elevated expression of zeb1, slug and/or twist1. Often, these TNFα-induced functions were connected to activation of the NF-κB pathway (77, 79, 80, 82, 83, 88).

Adding to reports describing the roles of TNFα in stemness/EMT induction in BC, a large number of studies indicate that IL-6 is a major inducer of stemness in this disease (72, 73, 89–96). IL-6 was found to promote stemness mostly in transformed but also in non-transformed breast epithelial cells. Many of these investigations have demonstrated the roles of IL-6 in promoting stemness by using a recombinant cytokine (73, 89–92); others have shown autocrine sources of IL-6, or demonstrated that the cytokine was released during tumor cell interactions with other cells (such as macrophages) (72, 73, 93, 95, 96). In line with the key roles of IL-6 in enhancing tumor cell stemness, the JAK-STAT3 pathway was connected to or proven to take part in these events (73, 90, 93–95); also, the Notch pathway was suggested to mediate IL-6-induced stemness in BC (91, 97).

### Inflammatory Chemokines Regulating Stemness and EMT

In addition to the pro-inflammatory cytokines TNFα and IL-6, chemokines such as CCL2, CCL5, and CXCL12 can regulate stemness and EMT in BC (reviewed in (50)). In parallel, strong impacts were found for ELR+ CXC chemokines in regulation of these aspects of plasticity in BC (50). Here, CXCL8 was demonstrated to act in autocrine and paracrine manners, depending on the research system used. The contribution of CXCL8 to stemness/EMT was demonstrated by determining the effects of recombinant CXCL8 (72, 74, 89, 98–100) and/or by using siRNA/neutralizing antibodies directed to the chemokine (74, 89, 96, 101–103). In such studies, CXCL8 was noted in CM of breast tumor/senescent cells or was induced by chemotherapy and by different stimuli such as over-expression of the transcription factor Brachyury (89, 96, 100, 101, 104). In parallel, several reports focused on the roles of the CXCL8 receptors CXCR1 and CXCR2 in controlling stemness and EMT in BC (99, 100, 104, 105). For example, inhibitors of CXCR1/2 reversed CXCL8-induced mammosphere formation by normal breast epithelial cells and by patient-derived BC cells (99, 105). In the latter study, the effect of the CXCL8–CXCR1/2 axis on stemness was shown to be through the transactivation of HER2 and EGFR by Src (105). Inhibitors of CXCR1/2 also showed that these receptor/s controlled the expression of EMT markers and induced elevations in tumor cell invasion (100, 103).

In parallel to CXCL8, CXCL1 also contributed to stemness (74, 106), but its impacts were mostly found in regulation of EMT-related processes, migration and invasion; in this respect, CXCL1 was found to act in an autocrine manner and/or to be secreted by macrophages in vicinity of the cancer cells (74, 76, 106, 107). Mechanistically, the involvement of several different signaling pathways in CXCL1-induced EMT processes was noted: NF-*κ*B (76), STAT3 (74) and also the MAPK pathway (107), proposing there is more than one molecular pathway linking CXCL1 stimuli with different EMT characteristics.

## Inflammation-Driven Therapy Resistance in Breast Malignancy

Therapeutic options given to BC patients depend on tumor subtype and clinical parameters, and their efficacy is often reduced because of intrinsic or acquired resistance. The molecular mechanisms enabling cancer cells to withstand chemotherapy, endocrine therapy, targeted therapy and immunotherapy are diverse and complex (2–4, 6). They represent, between others, selection of cells that underwent dynamic remodeling and gained the ability to subvert the effects of treatment (2–4, 6).

In the context of therapy resistance, inflammatory mechanisms are most influential and play key roles in shaping the nature and extent of therapy-relevant alterations taking place in the cancer cells (summarized in **Figure 1** and **Table 2**).

### Chemoresistance: Inflammation-Associated Myeloid Cells and Pro-Inflammatory Cytokines/Chemokines

Elevated levels of macrophages were detected in post-chemotherapy biopsies of BC patients compared to pre-chemotherapy samples; increased macrophage presence was also found in BC animal model systems following taxol treatment (108). In this research it was also demonstrated by *in vitro* studies that macrophage-derived cathepsins have protected the tumor cells from taxol-induced cell death, and that cathepsins also reduced the efficacy of taxol *in vivo* (108). In another research, macrophages were connected to chemoresistance when antibodies targeting colony-stimulating factor-1 (CSF-1), a major monocyte chemoattractant, increased the efficacy of chemotherapy in reducing tumor sizes *in vivo*, in a process that was accompanied by lower macrophage presence in tumors (109). Phenotypically, it was found that M1 macrophages secreted factors that have led to elevated proportions of ALDH+ CSCs through NF-*κ*B and STAT3, and *via* the Lin-28B-let-7-HMGA2 axis; these CSCs expressed increased resistance to doxorubicin, 5-FU and paclitaxel (73).

In parallel, another study demonstrated key roles for the CD11b+Gr1+ myeloid sub-population in mediating resistance to chemotherapy in BC. These cells were recruited to tumors by cancer cell-derived CXCL1/2 chemokines, and served as a major source for the pro-inflammatory proteins S100A8 and S100A9. In turn, S100A9 was found to be responsible for increased survival of the cancer cells in mice treated by doxorubicin and cyclophosphamide (110). Accordingly, analysis of BC patient biopsies demonstrated increased expression of S100A8/9 following chemotherapy (110). The findings of this study also indicated that the CXCL1/2–S100A8/9 axis was reinforced by chemotherapy through a stroma-derived TNFα-mediated process (110). TNFα roles in inducing therapy-resistant breast CSCs were further demonstrated when it was found that extended exposure of BC cells to combined TNFα+TGFβ stimulation has led to generation of CSCs that acquired high levels of resistance to chemotherapy, accompanied by elevated expression levels of ABC transporters (84).

Being a part of the complex network of TNFα and its ligands, the transmembrane form of TNFα (tmTNFα) was found to be expressed at high levels in a considerable proportion of BC patient tumors, mainly of the TNBC subtype (111). By using primary tumor cells, it was demonstrated in this study that high expression levels of tmTNFα were correlated with elevated resistance to anthracycline (111). Making use of shRNA to TNFα and the N-terminal fragment of tmTNFα, the authors of this investigation have concluded that reduced expression of tmTNFα improved the sensitivity of breast tumor cells to doxorubicin, and that the activities of tmTNFα in resistance were meditated by NF-*κ*B and Erk (111). In addition, a recent study concerning the TNFα receptor TNFR2 in TNBC and luminal-A BC cells indicated that its down-regulation has improved the efficacy of adriamycin; TNFR2-mediated resistance was taking place through inhibition of adriamycin-induced pH2AX expression, mediated by enhanced expression of poly(ADP-ribose) polymerase (PARP) (112).

Throughout these investigations, some insights were provided to molecular mechanisms that may mediate inflammation-driven processes of chemoresistance. Here, major roles were attributed to the transcription factor NF-*κ*B (103, 111, 113, 114). Particularly, a recent study that has analyzed cross-tolerance of breast tumor cells to anthracyclines (doxorubicin), which has developed in taxane (doxetaxel)-resistant cells, indicated that inflammatory cytokines and NF-*κ*B were involved in the process (114); this investigation has indicated that GM-CSF + IL-23 + IFN*γ*, as well as NF-*κ*B-mediated signaling induced the expression of CD44 by taxane-resistant cells, initiating signaling and metabolic cascades that regulated cross-tolerance (114). In addition, STAT3 was strongly implicated in reducing the sensitivity of breast tumor cells to chemotherapies. In line with the fact that STAT3 is a key transcription factor mediating the effects of IL-6 (42, 115), direct roles were reported for IL-6 in inducing chemoresistance, which was mediated by activation of C/EBP (116).

However, irrespectively of IL-6 activities, STAT3 involvement in chemotherapy resistance was proposed by a number of publications. For example, in doxorubicin-resistant TNBC cell inhibition of STAT3 activation by pharmacological inhibitor partly restored cancer cell sensitivity to the treatment, possibly through reduction in CSC proportion (117). In another study it was found that IL-22—whose expression by T cells was increased in TNBC tumor tissues compared to para-tumor and normal areas—induced paclitaxel resistance in TNBC cell lines, accompanied by elevated JAK/STAT3 activation (118). The JAK/STAT3 pathway was also found to regulate leptin-induced fatty acid β-oxidation, promoting self-renewal and chemoresistance in breast CSCs (119). This latter study also reported that STAT3 mRNA levels were higher in *ex vivo* cultured BC tumors derived from post-chemotherapy TNBC biopsies compared with pre-chemotherapy tumor tissues (119).

As part of the mechanistic analyses included in the above studies, the chemokines CXCL1/2 were found to be involved in regulation of chemoresistance (110). The roles of such chemokines in this process are strongly supported by additional investigations addressing inflammatory ELR+ CXC chemokines and their receptors in inducing or mediating lower sensitivity levels to chemotherapies. Different studies indicated that various types of chemotherapy share the ability to promote the release of ELR+ CXC chemokines—CXCL1, CXCL2, CXCL3, CXCL5, CXCL7 and CXCL8 (depending on the study) —by myeloid cells, breast tumor cells and mesenchymal stem cells (103, 104, 110, 120, 121). In parallel, inhibition of CXCL8 or of CXCR1/2 has increased the sensitivity of breast tumor cells to chemotherapeutic drugs, accompanied by reduced proportion of CSCs, angiogenesis, tumor growth and/or metastasis, with roles attributed to cyclooxygenase 2 (COX-2) in this process (103, 104, 110, 113, 121, 122). These findings have a high clinical relevance in view of the fact that chemotherapy has led to increased CXCR2 expression in BC patients; moreover, increased CXCR1/CXCR2 and CXCL8 expression levels were significantly correlated with poor overall and disease-free survival in studies of the TCGA dataset and of patient biopsies (103, 122).

### Resistance to Other Therapies: Inflammation-Associated Myeloid Cells and Pro-Inflammatory Cytokines/Chemokines

In a way similar to chemotherapy, recent lines of evidence indicate that inflammatory components controlled also resistance to other types of therapy in BC. For example, TNFα and/or IL-6 were strongly connected to endocrine resistance in luminal-A breast tumors, in patients as well as in model systems of cultured cells or mice (123–126). The study of tumors of ER+ HER2- BC patients revealed that resistance to tamoxifen was significantly associated with the presence of CD163+ macrophages in tumors (123). High macrophage counts were also connected to poor outcome in ER+ patients in other studies (125, 127) and macrophages were found to release factors that promoted endocrine resistance, such as TNFα; here, TNFα acted by inducing down-regulation of FOXO3a, leading to ERα down-regulation (125). In another study, TNFα was found to induce in macrophages the release of factors that have led the cancer cells resist estrogen withdrawal and express elevated resistance to tamoxifen and ICI 182,780; co-culture experiments of macrophages with cancer cells demonstrated that TNFα together with IL-6 have led to increased activation of STAT3, NF-*κ*B and ER*α*, thus leading to its constitutive stimulation (124).

Moreover, macrophages and CCL2 were correlated with each other and with poor survival in ER+ patients; it was also found that monocytic cells cultured with CM of tamoxifen-resistant luminal-A cells secreted elevated levels of CCL2, which then acted directly on BC cells to increase endocrine resistance *via* the PI3K/AKT/mTOR pathway (127). Along the same lines, the inflammatory chemokine CCL5 acted in autocrine manners to induce STAT3 activation, leading to tamoxifen resistance (128).

Roles for macrophages and inflammatory cytokines/chemokines were also proposed in resistance of tumor cells to measures targeting HER2 and other receptor tyrosine kinases (129–131); there are also indications of similar roles for inflammatory cells/mediators in resistance to immunotherapy. In this specific case, the situation is even more complex because of the feedback mechanisms that dictate the equilibrium between acquired immunity and the inflammatory arm of the immune system. Here, it was demonstrated that the efficacy of immunotherapies can be repressed by myeloid cells and regulatory lymphocytes as well as by soluble pro-inflammatory mediators. It was found that MDSCs and regulatory T cells (Tregs) interfered with the beneficial effects of immunotherapies and have led to immune plasticity that was strongly connected to immune resistance (132–139). Moreover, co-targeting inflammatory pathways alongside with the use of immune checkpoint blockades has led to improved efficacy of immunotherapies (140–145).

## Inflammation-Driven Tumor Cell Dormancy in Breast Malignancy

Dormancy is another level of cancer cell plasticity that reflects the dynamic nature of disease progression, having major implications on recurrence-free rates in patients (2, 8). Dormant tumor cells are recognized by a temporary mitotic arrest, leading to a viable but non-proliferating cell state (6). Early dormancy may take place in primary tumors, but often dormancy is observed following cancer cell spreading to metastatic sites. Disseminating tumor cells that have entered dormancy can evolve and escape this state, the result being tumor relapse and disease recurrence (4, 6–8).

The mechanisms controlling entry to and escape from dormancy are currently being extensively studied. In some of the experimental systems described below, dormancy was investigated by using variant tumor cells that remained undetectable and re-emerged after a considerable long latency; in other studies, cancer cells that have entered dormancy following chemotherapy or other manipulations became proliferative and led to tumor recurrence.

It is now clear that lymphocytes such as T helper (Th) cells and CTLs establish a hostile microenvironment to the cancer cells (23, 24). Immunologic dormancy was evidenced in many tumor systems, manifesting the fact that immune mechanisms have considerable roles in determining whether the cancer cells will enter dormancy and stay quiescent, or if they will become fully equipped with the machineries that enable them to re-emerge and metastasize (17, 18, 23, 24). If this stage takes place when beneficial aspects of acquired immunity are suppressed or when the patient is not treated by tumor-limiting drugs, exit from dormancy would serve well the needs of the tumor cells and lead to recurrence and disease progression.

The activities of key immunological players like effector Th1 cells and CTLs take place alongside inflammatory processes that recently have been identified as a leading force in promoting escape from dormancy. Specifically in BC, a strong connection was revealed between inflammatory conditions and tumor cell exit from dormancy, metastasis and recurrence. This has been demonstrated under conditions like obesity-associated inflammation or inflammation induced by exposure to tobacco smoke or to lipopolysaccharide (146–148). With respect to obesity, it was found by deGraffenried and colleagues that disease recurrence was significantly reduced in ERα+ obese patients upon use of nonsteroidal anti-inflammatory drugs (NSAIDs), which are potent inhibitors of inflammation (148).

Links were also recently made between surgery/wound healing and enhanced emergence from dormancy (149–155). This path was then connected to inflammation in BC, for example by a study addressing surgery- and chemotherapy-induced dormancy (156). This research has demonstrated that administration of ketorolac—which is an analgesic with NSAID activities targeting COX-1 and COX-2—reduced awakening from dormancy and tumor recurrence in a Lewis lung cancer (LCC) model, and that the dormancy process was mediated by COX-1 (156). Connecting these findings to BC is the fact that similar to LCC, pre-operative ketorolac administration prolonged animal survival after mastectomy in a TNBC model system (156). Furthermore, intra-operative administration of ketorolac to BC patients significantly prolonged disease-free survival (155).

Within the scope of the close connections between inflammation and dormancy, the following observations were made (summarized in **Figure 1** and **Table 3**).

### Inflammation-Associated Myeloid Cells Regulating Tumor Cell Dormancy

The roles of macrophages in regulating dormancy were demonstrated in a recent study addressing wounds formed in an immunogenic model of BC (157). In this research it was found that BC cells devoid of metastatic capabilities gained the ability to disseminate and grow in remote organs after surgery. Moreover, surgical wounding has induced a systemic inflammatory response and accordingly, administration of the NSAID meloxicam, starting prior to surgery, had an inhibitory impact on tumor growth (157). The inflammatory reaction was manifested by elevated levels of circulating myeloid cells (monocytes and neutrophils), and elevated expression of inflammatory mediators, including the major monocyte chemoattractant CCL2; CCL2 down-regulation has led to partial reduction in tumor outgrowth (157).

These findings emphasize the roles of myeloid cells in dormancy control in BC. Here, it is interesting to note that breast tumors were found to be enriched with a M2-related macrophage subset that was localized in proximity to blood vessels in primary tumors and bone metastases of BC patients (158). Animal studies demonstrated that these macrophages expressed vascular endothelial growth factor A (VEGFA) and that following chemotherapy, tumors lacking VEGFA+ macrophages recurred in slower kinetics than tumors containing VEGFA+ macrophages (158).

As mentioned above, COX-1 and COX-2 were proposed as pro-inflammatory mediators that enhanced awakening tumor cells from dormancy (148, 156). The above-mentioned study by the deGraffenried group also demonstrated the close relations of COX enzymes to macrophages in context of dormancy (148). Here, sera of obese ERα+ patients induced the expression of COX-2 in macrophages, leading to greater aromatase expression by pre-adipocytes (148); then, aromatase that was released by macrophages/pre-adipocytes grown with sera derived from obese patients, induced in ERα+ breast tumor cells the activity of ERα, tumor cell migration, and proliferation (148).

Another link connecting dormancy and macrophages was made by showing the involvement of chemokines that recruit monocytes, such as CCL2 and CCL5, in such processes. Roles for CCL2 in driving forward monocyte recruitment, then leading to exit from dormancy, were discussed in the context of wound healing, as mentioned above (157). In parallel, in a research system based on HER2 down-regulation that has led to generation of residual tumors and then to tumor recurrence, CCL5 induced elevated presence of macrophages that promoted emergence from dormancy. This effect was mediated by increased presence of CCR5+ collagen-depositing macrophages in residual tumors and CCL5 over-expression in tumor cells has led to faster recurrence (159). Moreover, this CCL5 study (159) also has made an interesting connection between dormancy and TNFα activities, through the NF-*κ*B pathway. It has shown that HER2 down-regulation has induced a pro-inflammatory program that included TNFα, which through activation of the IKK-NF-*κ*B pathway has given rise to chemokine induction; the chemokines included CCL5 that mediated the increased abundance of CCR5+ macrophages, which contributed to escape form dormancy (159).

### Pro-Inflammatory Cytokines Regulating Tumor Cell Dormancy

The above studies provided evidence to roles of macrophage-associated cytokines and chemokines in controlling cancer cell dormancy. Alongside with TNFα that was reported above, other strong pro-inflammatory cytokines were found to regulate dormancy, for example by increasing the proliferation of dormant cancer cells at a bone-like microenvironment. In a study addressing not only TNFα but also IL-1β, the two cytokines were shown to increase tumor cell proliferation in a metastasis-suppressed model of a TNBC cell line and in combination with IL-6 and CXCL8 also of luminal-A cells (160). This study has demonstrated that the NSAID indomethacin and a prostaglandin E2 (PGE2) antagonist inhibited the pro-tumor effects of the cytokines (160), thus joining other reports that provided evidence to major roles for COX enzymes and PGE2 in promoting exit from dormancy [*e.g.*, (148, 156)].

In parallel, it was found that IL-6 supported the growth of luminal-A BC cells in “dormant colonies”, but lowered the proliferation of the same cells in “growing colonies” (161). These findings suggest that IL-6 may potentially have opposing roles in control of tumor cell growth and dormancy, depending on intrinsic properties of the cancer cells.

It is interesting to note that another member of the IL-6 family, leukemia inhibitory factor (LIF) was found to maintain the dormancy state in breast tumor cells (162), being in line with previous studies suggesting that LIF receptor (LIFR) is a tumor suppressor gene [*e.g.* (163)]. Low mRNA levels of LIFR and its downstream signal transducer STAT3 were significantly associated with bone metastasis and poor prognosis, respectively, in BC (162). This observation was followed up by *in vitro* tests that linked LIFR down-regulation to increased migration and invasion abilities of luminal-A tumor cells; moreover, LIFR knockdown has led to increased proliferation of the tumor cells and elevated osteoclastogenesis/bone destruction in mice, in contrast to WT cells that remained in a dormant phenotype (162). Of note is the fact that low metastatic cells that migrated to the bone niche and stayed in a dormant state were sensitive to the effect of LIF : LIFR on the activation of STAT3, while TNBC cells that normally did not enter dormancy did not respond to LIF stimulation (162).

### Inflammatory Chemokines Regulating Tumor Cell Dormancy

Some of the studies mentioned above addressed also CXCL8, demonstrating that it could promote escape from dormancy on one hand (160, 161), but it reduced the proliferation of breast tumor cells in “growing colonies” (161). Here, it is important to indicate that in contrast to the above-mentioned findings on CXCL8-mediated down-regulation of BC cells at growing phase (161), CXCL8 and other members of the ELR+ CXC sub-group of chemokines are generally and largely considered as key promoters of cell proliferation and viability in BC [reviewed in (50)]; thus, it is assumed that such chemokines would often enhance re-emergence from dormancy.

Supporting this view is a recent study addressing the hepatic niche in which BC cells often colonize (164). This report has demonstrated that CXCL8 was released by hepatic stellate cells and has increased TNBC cell proliferation through its receptor CXCR2 and ERK signaling activation; this pathway was relevant to dormancy because CXCL8 contributed to emergence from doxorubicin-induced dormancy in an *ex vivo* 3D liver micro-physiological system (164). In another report, the ELR+ CXC chemokine CXCL5 was shown to up-regulate BC cell proliferation in an *ex vivo* tumor-bone co-culture system that assessed the switch from dormancy to colonization through stimulation of tumor cell growth (165). In that investigation, CXCL5 expression levels were elevated in co-cultures using bones derived from cancer-bearing mice compared to bones from healthy mice; it was also found that CXCL5 increased murine breast tumor cell proliferation under quiescent conditions and that the process was mediated through CXCR2, whereas factors derived from bones of healthy mice induced cancer cell quiescence (165).

## Final Considerations and Perspectives

Extensive research has led scientists and physicians to realize that cancer cell plasticity puts its marks on tumor fate and dramatically influences disease course in malignancy. Tumor cell remodeling stands in the basis of phenotypic and functional heterogeneity and leads to constant changes in tumor characteristics, thus having cardinal clinical implications (4, 5, 9, 10).

In this review, addressing breast cancer as a representative model system, we have demonstrated that pro-inflammatory constituents enhance cancer cell plasticity by increasing stemness/EMT, therapy resistance and exit from dormancy. These three remodeling forms can be driven and up-regulated by similar pro-inflammatory elements: inflammation-associated myeloid cells, pro-inflammatory cytokines and inflammatory chemokines, as indicated in the papers summarized in this review and summarized in **Figure 1** and **Tables 1**–**3**. Moreover, the canonical transcription factors that mediate the functions of some of the mediators, namely NF-*κ*B and STAT3, were generally implicated in generating tumor cell plasticity or maintaining it at different stages of the malignancy cascade (35, 39, 40, 115).

In this manuscript, we have portrayed an interactive remodeling scenario, in which three forms of plasticity are connected, with inflammation being a common thread that links them all. These observations set inflammation as a potential target in cancer therapy. To give an example, one therapeutic approach could be to use inflammation to trigger the awakening of tumor cells from dormancy, but to do so in a well-controlled manner that allows “hitting” the cancer cells when they enter a proliferating step, *e.g.*, by chemotherapy. However, not only that “reviving” the tumor is a risky step, but also one should take into consideration the possibility that inflammatory processes may halt mechanisms of immune surveillance that could have been beneficial in fighting the re-arising tumor.

The opposite approach could take advantage of the fact that inflammation forms a regulatory hub in promoting tumor cell plasticity, setting inflammation as a prime target for therapy whose inhibition may reduce stemness/EMT, therapy resistance and dormancy, all at the same time. Indeed, as inflammation seems to be a common denominator that strongly connects various aspects of tumor cell remodeling, inhibition of inflammatory elements may reduce a number of cell remodeling processes simultaneously.

In this context, measures that combine efforts to reduce inflammation and in parallel strengthen anti-tumor immune activities (*e.g.*, immunotherapies) (as exemplified in (140–145)), may have an even greater benefit in the clinic. This approach, that tilts the immune balance by down-regulating inflammation and increasing protective immunity, is practically feasible. For example, in the clinic, TNFα inhibitors are used with relatively high success in therapy of autoimmune diseases and inflammatory disorders, and therapies directed to IL-6 are used in rheumatoid arthritis (166–168). In parallel to the potential use of TNFα and IL-6 inhibitors, one can also consider reducing the recruitment of monocytes to primary tumors and metastases, *e.g.*, by inhibiting the activities of chemokines and their receptors, as well as of other cytokines that support monocyte migration and macrophages activities, like CSF-1 (49, 52, 143, 169). These measures can be used together with immunotherapies that have been already introduced to patients in different types of malignancies, including current clinical trials in TNBC [*e.g.* of antibodies directed to PD-L1 (170)].

Obviously, it is not an easy task to tune the equilibrium in favor of anti-tumor activities and more efficient immune surveillance; moreover, one should consider the possibility that specific immune/inflammatory mediators or transcription factors may have opposing roles depending on intrinsic and extrinsic signals. One example to such a problematic scenario was described above, when STAT3 was found to have key roles in promoting cancer cell remodeling, *e.g.*, following IL-6 activation, but in response to LIF activation kept dormant BC cells in check (42, 115, 171).

These aspects emphasize the need to carefully identify the inflammatory mechanisms regulating tumor cell plasticity. Thus, to successfully implement a combined tactic of inhibiting inflammation while promoting protective immunity, a detailed and accurate analysis of the elements involved in driving and regulating tumor cell remodeling should be performed in each and every cancer type and subtype.

## Author Contributions

TB, LR-A, and HB-Y contributed to literature search, manuscript writing and editing. HB-Y also assisted in figure preparation. AB-B was responsible for the entire setup and structure design of the manuscript, and participated in all other aspects of article preparation including writing, editing, and figure design. All authors contributed to the article and approved the submitted version.

## Conflict of Interest

The authors declare that the research was conducted in the absence of any commercial or financial relationships that could be construed as a potential conflict of interest.
